# Prevalence of Winter Ticks (*Dermacentor albipictus*) in Hunter-Harvested Wild Elk (*Cervus canadensis*) from Pennsylvania, USA (2017–2018)

**DOI:** 10.3390/vetsci7040177

**Published:** 2020-11-12

**Authors:** Elizabeth Calvente, Samantha Pelletier, Jeremiah Banfield, Justin Brown, Nicole Chinnici

**Affiliations:** 1 Dr. Jane Huffman Wildlife Genetic Institute, University of Pennsylvania, East Stroudsburg, PA 18301, USA; spelletier@everence.life (S.P.); nchinnici@esu.edu (N.C.); 2 Pennsylvania Game Commission, 2001 Elmerton Avenue, Harrisburg, PA 17001, USA; jebanfield@pa.gov; 3 Department of Veterinary and Biomedical Sciences, Penn State University, University Park, PA 16802, USA; jdb56@psu.edu

**Keywords:** *Cervus canadensis*, *Dermacentor albipictus*, *Ixodes scapularis*, hunter-harvested elk, Pennsylvania, prevalence, tick, winter tick

## Abstract

Winter ticks (*Dermacentor albipictus*) are an aggressive one-host tick that infest a wide-diversity of ungulates. Infestations can result in anemia, alopecia, emaciation, and death. Most notably, the winter tick has caused negative impacts to moose (*Alces alces*) populations in the northeast United States and Canada. Winter ticks have been identified on other cervid species, including deer (*Odocoileus virginianus*) and elk (*Cervus canadensis*), which generally results in low tick burdens and mild or no disease. Recently, however, a wild yearling bull elk in Pennsylvania was found dead as a result of severe winter tick infestation. To obtain baseline data on winter ticks in wild elk in Pennsylvania, we collected 1453 ticks from 190 hunter-harvested wild elk between 2017–2018. Of the 204 harvested elk, 94.3% (190/204) had ticks collected for this study and none of the sampled elk had evidence of winter-tick associated disease. The average tick burden was 7.7 ticks/elk and average winter tick load on all elk was 0.5. Results of this study indicate that winter ticks do infest wild elk in Pennsylvania. However, during the fall months, the tick burden is low and rarely associated with lesions. These data herein serve as a baseline to monitor winter tick populations over time.

## 1. Introduction

Winter ticks (*Dermacentor albipictus*) can negatively impact the health of cervids directly through heavy infestations and indirectly through transmission of multiple pathogens. Classic symptoms of winter tick infestation include excessive grooming and premature loss of winter hair [1], anemia and late winter mortality in calves [2], as well as weight loss, shedding of winter coat, and death [3]. Common pathogens transmitted by winter ticks include *Anaplasma marginale* and *Babesia duncani* [4,5]. Although there have been no recorded cases of *B. duncani* infection in elk, *Babesia odocoilei* can cause severe Babesiosis characterized by hematuria, lethargy, and death [6]. Additionally, elk have been shown to develop asymptomatic infections after experimental infections with *A. marginale* [7]. *Anaplasma marginale* transmission by winter tick and other *Dermacentor* species has been reported in ungulates including cattle (*Bos Taurus*), mule deer (*Odocoileus hemionus*), and big horned sheep (*Ovis canadensis*) with bovine anaplasmosis having a negative impact on the US cattle industry [8,9,10,11].

The winter tick life cycle occurs over six months on a single host, beginning in early fall (September–October) when larval ticks infest cervids. Parasitic periods (time spent by the tick in each life stage before molting to the next) can fluctuate in length under varying conditions. In colder climates, larval winter ticks will molt into nymphs and enter a diapause before becoming active again in mid-January. Nymphs will feed and molt into adults from mid-January to March and in April, engorged females will drop-off [12]. A recent health survey of Boreal Caribou (*Rangifer Tarandus caribou*) found winter tick associated hair loss in 81% of the surveyed population during winter months [13]. Infestations by winter ticks have proven to be especially devastating to moose (*Alces alces*) populations in the northeast United States and Canada resulting in anemia, excessive grooming, damage to the skin, hair loss, emaciation, and ultimately death [1,2,3]. While moose are susceptible to severe winter tick infestation, other cervid species such as elk (*Cervus canadensis*) experience programmed grooming which assists in removing ticks before they attach and feed, preventing severe infestation and reducing disease spread [14,15,16]. Although rare, cases have been documented showing similar health implications of winter tick infestations on elk in Wyoming [17]. Similarly, in 2017, mortality associated with severe winter tick infestation was recently documented in a wild yearling bull elk from Pennsylvania (PA) [18].

Warmer temperatures in Autumn months and delays in snow fall extend time larval winter ticks can quest, potentially increasing their abundance on ungulates. Additionally, shorter and milder winters promote adult female tick survival and increase egg survival in early summer [19]. In New Hampshire, increases in epizootic events caused by winter tick infestations on moose is thought to be associated with shorter and milder winters [20,21,22]. Similar changes in Pennsylvania autumn and winter months make it necessary to develop baseline data on winter tick populations to determine risk of potential epizootic events in the future.

The intent of this study was to identify the presence of winter ticks on wild elk in Pennsylvania. The most opportune time to achieve this is during the annual elk hunt in October and November from hunter-harvested elk. This sampling approach limits our ability to characterize temporal changes influencing tick diversity on Pennsylvania wild elk due to the variation in presence or absence of tick species throughout each season of the year. Therefore, the information in this study should not be interpreted as a complete picture of the tick species present on wild elk in Pennsylvania but only as the confirmation of winter tick presence in general.

In this report, we characterized the prevalence, diversity, and burden of ticks collected from 190 wild elk during the 2017 and 2018 elk hunt in Pennsylvania, which occurs during early November.

## 2. Materials and Methods

### 2.1. Tick Collection and Storage

In 2017 and 2018, ticks were collected during the 6-day hunting season in early November from wild elk harvested in five counties in Pennsylvania, USA; Elk county, Clearfield county, Cameron county, Centre county, and Clinton county. The carcass of every harvested wild elk in Pennsylvania must be delivered to the check station within 24 h of when it was killed. At the check station, the entire elk carcasses were examined by Pennsylvania Game Commission (PGC) personnel for 3–5 min and any ticks observed were collected and stored in whirl-packs. Sampling time was implemented for practical purposes to account for the large number of elk brought to the check stations. Ticks collected by this technique represent a small subsample of the total population of tick on elk. Additional information collected from sampled elk included location and date of harvest, sex, age (based on tooth histology), body condition, and any grossly apparent lesions in the skin or hair coat. Collected ticks were stored at −20 °C until they were identified at the Dr. Jane Huffman Wildlife Genetics Institute (East Stroudsburg, Pennsylvania).

### 2.2. Tick Identification

Tick identification was performed at the Dr. Jane Huffman Wildlife Genetics Institute. Based on morphological characteristics; the species, sex, and life stage were identified for each collected tick [23]. Species of tick was confirmed with PCR and sequencing of the 16s rRNA mitochondrial gene using the 16s+1 rRNA primer (5′CCGGTCTGAACTCAGATCAAGT3′) from Krakowetz et al. as the forward primer for PCR and sequencing and the 16s+1 rRNA primer (5′CTGCTCAATGATTTTTTAAATTGCTGT3′) from Nadolny et al. as the reverse primer for PCR and sequencing [24,25]. Using a Qiagen DNeasy^®^ Blood and Tissue Kit (Qiagen Sciences Inc., Germantown, MD, USA), DNA was extracted from the legs of each tick [26]. Amplification was performed in a 20 µL reaction consisting of 1X Promega^TM^ Go Taq^TM^ Master Mix (Promega Corporation, Fitchburg, WI, USA), dH2O, 1 µM forward and reverse primer stock, and purified DNA. Thermal cycler conditions were performed with an initial denaturation of 95 °C for 5 min followed by 40 cycles of 95 °C for 30 s, 55 °C for 45 s, 72 °C for 45 s, with a final extension of 72 °C for 5 min. Following PCR amplification, all PCR products were visualized using a 1% agarose gel stained with ethidium bromide. A post-PCR clean-up was performed using ExoSAP-IT and base pair sequences were generated using the BigDye Terminator v3.1 Cycle Sequencing Kit and an Applied Biosystems 3130 genetic analyzer (Applied Biosystems, Foster City, CA, USA). Morphologically identified winter ticks were confirmed via sequencing (GenBank Accession: AY676458.1).

### 2.3. Statistics

Statistical analysis was performed using IBM SPSS^®^ Statistics for Windows version 24.0 (IBM, 2016). Significant differences between compared groups indicate a measurable inequality of observed data. A chi-square analysis was performed to compare male to female ratios of both tick species combined and ratios within each species. A comparison of adult and nymph ticks was also performed with a chi-square analysis. A one-way ANOVA of total tick load per elk per county, of blacklegged tick load per elk per county, and of winter tick load per elk per county was performed to compare the tick populations within each county without the influence of the difference in number of elk harvested per county. An alpha of 0.05 was used to determine statistical significance.

## 3. Results

**Figure 1:**
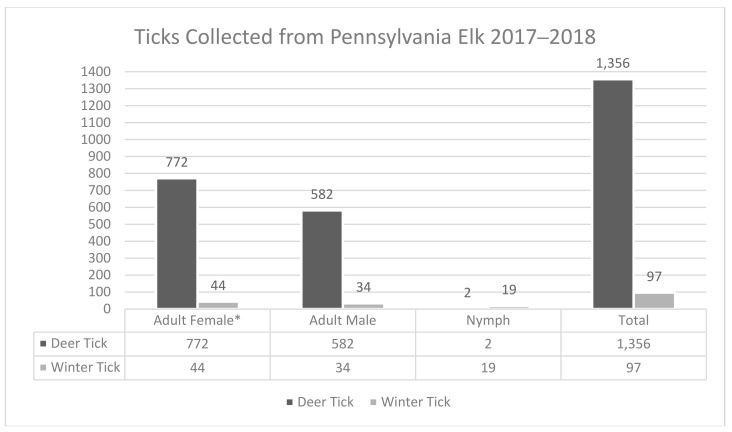
Total number of ticks collected from Pennsylvania elk during the 2017 and 2018 elk hunt. Data is separated by species, sex, and life stage. * Significance of less than 0.05 to adult males.

Overall, 1453 ticks were collected from a total of 190 elk during the 2017 (99 elk; 931 ticks) and 2018 (91 elk; 522 ticks) Pennsylvania elk hunts (Table 1). None of the elk examined had any skin lesions or pelage damage consistent with severe winter tick infestation. Two tick species were identified; *Ixodes scapularis* (blacklegged) ticks (n = 1356) and winter ticks (n = 97) (Figure 1). Both species of tick were found on 21.1% (40 out of 190) of elk, whereas 1.7% (three out of 190) and 77.4% (147 out of 190) of elk had only winter ticks or only blacklegged ticks, respectively. Of the 190 elk, 43 were identified to have a minimum of one winter tick with an average winter tick load of 2.3 (Table 2).

In 2017, ticks were identified and collected from 94.3% (99 out of 105) of harvested wild elk. Of these, 23.8% (25 out of 105) had winter ticks (n = 63) and 93.3% (98 out of 105) had blacklegged ticks (n = 868). In 2018, ticks were identified and collected from 91.9% (91 out of 99) of harvested wild elk. Of these, 18.2% (18 out of 99) had winter ticks (n = 34) and 89.9% (89 out of 99) had blacklegged ticks (n = 488).

Male and female winter tick populations were evaluated using a chi-square for 2017 (*x*^2^ = 0.087; *p* = 0.768), 2018 (*x*^2^ = 2.000; *p* = 0.157) and with data combined (*x*^2^ = 1.282; *p* = 0.258). Adult and nymph winter tick populations were evaluated using a chi-square for 2017 (*x*^2^ = 13.349; *p* = 0.0003) and 2018 (*x*^2^ = 26.471; *p* = 0.0000003). Male and female populations of blacklegged ticks were evaluated using a chi-square for 2017 (*x*^2^ = 13.079; *p* = 0.00006) and 2018 (*x*^2^ = 10.623; *p* = 0.001) and with data combined (*x*^2^ = 26.662; *p* = 0.0000002). None of the sampled elk had evidence of winter-tick associated disease.

Ticks were collected from elk that were harvested from five counties, namely Elk, Clinton, Cameron, Centre, and Clearfield. Total tick distribution per county was evaluated using a one-way ANOVA. A post hoc Tukey HSD showed statistical significance in tick load per elk between Elk and Clearfield (95% likely range 0.1731–1.556, *p* = 0.007), Elk and Cameron (95% likely range 0.3900–1.816, *p* = 0.0004), Elk and Clinton (95% likely range 0.1298–2.1518, *p* = 0.019), and Elk and Centre (95% likely range 0.3244–2.4639, *p* = 0.004) counties in 2017. In 2018, there was significance between Elk and Cameron (95% likely range 0.1848–5.4224, *p* = 0.030) and Clearfield and Cameron (95% likely range 0.2234–6.5266, *p* = 0.030). When combining data sets from both years, a significant difference was found between Elk and Cameron (95% likely range 0.3984–1.3867, *p* = 0.00002) and Elk and Centre County (95% likely range 0.2488–1.6371, *p* = 0.002) (Table 1).

Total winter tick distribution per county varied with 26.8% (26 out of 97) collected from Elk County, 27.9% (27 out of 97) from Clearfield County, 16.5% (16 out of 97) from Cameron County, 15.5% (15 out of 97) from Centre County, and 13.4% (13 out of 97) from Clinton County (Table 1). There was a significant difference in winter tick load per elk between counties in 2018 (95% likely range 1.0210–2.7568, *p* = 0.0003). However, a post hoc test could not be run because at least one group had fewer than two cases (Table 1).

Blacklegged tick distribution per county was evaluated for 2018 (95% likely range 4.8482–6.2978, *p* = 0.006) and both years combined (95% likely range 6.3622–8.0420, *p* = 0.037). A post hoc Tukey HSD showed a statistical significance in blacklegged tick load per elk between Elk and Cameron (95% likely range 0.0055–5.3631, *p* = 0.049) and Clearfield and Cameron (95% likely range 0.04344–6.7200, *p* = 0.017) County for 2018, and between Elk and Cameron County (95% likely range 0.4464–6.7820, *p* = 0.016) when data were combined.

## 4. Discussion

In this study, we collected ticks from harvested wild elk in Pennsylvania during the 2017 and 2018 elk hunting season and identified them to the species level. A total of 1453 ticks were collected from 190 harvested elk. Blacklegged ticks and winter ticks were the only two species that were identified during this survey. Both species of tick were found on 21.1% (40 out of 190) of elk, whereas 1.7% (three out of 190) and 77.4% (147 out of 190) of elk had only winter ticks or only blacklegged ticks, respectively. These results are consistent with a previous study on wild white-tailed deer (*Odocoileus virginianus*) from Pennsylvania during November and December, which only identified blacklegged ticks and winter ticks. Blacklegged ticks were found to be more abundant and widespread than winter ticks in this study as well [27].

Our tick survey and that performed by Baer-Lehman et al. (2012) relied on sample collection from hunter-harvested cervids [27]. This sampling approach allows for large numbers of animals to be sampled; however, one limitation is that the timing of collection is restricted to the open season, which for cervids is typically in the late fall or winter. The timing of this sampling strongly influences tick prevalence results, as tick species and abundance are seasonally dependent. While the results reported herein can be used to identify distribution of winter tick and black-legged tick, based on their life cycle, there are many other tick species (i.e., American dog tick, *Amblyomma americanum*) that are not present on the host in the Fall and consequently would not be identified. For example, a tick surveillance study conducted by Slabach et al. (2018) surveyed 1617 ticks collected from 255 wild elk from Kentucky over three years, predominately during spring elk captures, and the most common tick species identified were 52.3% (845 out of 1617) winter ticks and 42.1% (680 out of 1617) *Dermacentor variabilis* (American dog ticks) [28]. Deer ticks were also identified in the Kentucky study but only 0.9% (15 out of 1617) of the total due to their reduced presence during the spring months. We did not identify American dog ticks in our study, despite their presence in Pennsylvania, because this tick is not present on hosts during the time of elk hunting season in Pennsylvania. Tick species vary geographically and seasonally resulting in difference in dominance of tick species, overall tick load, and potential disease impacts on host. In the northeast, the most common tick species during the fall is the adult blacklegged tick, while other tick species such as *Amblyomma americanum* and American dog ticks are dormant [29,30].

Statistical analyses of male to female ratios, nymph to adult ratios, and tick load per elk per county were performed to better characterize the tick population. Chi-square analysis found statistical differences between male and female ticks in blacklegged tick populations for 2017, 2018, and with data combined. Significant differences were also found between adult and nymph winter tick populations in 2017 and 2018. Data from these analyses indicate female ticks contributing more significantly to the overall population than males. These results are important when considering the potential for population changes in the future. A higher population of female ticks will result in more eggs and larval ticks in the spring, increasing the population in the next season. High female populations also increase the risk for disease transmission as female ticks will attach and feed for longer periods of time. A one-way ANOVA analysis compared the tick populations per county without the bias of differences in number of harvested elk per county by comparing tick load per elk per county. A Tukey HSD analysis determined a significance in ticks collected from Elk county for 2017, 2018, and both years combined, suggesting a higher population of ticks within this county. Results from these analyses can assist with targeted wildlife management or surveillance of specific counties and subsets of elk within the total population to continue monitoring the parasitic load.

Beginning in August, larval winter ticks will attach to hosts and molt into nymphs within 10–22 days. Differences in rate of parasitic development of winter tick nymph have been documented in various studies. Winter tick nymphs in colder climates with more severe winters will enter a diapause in the fall lasting up to three months before molting into adults in January. Time from nymph to adult can range from 41 to 200 days [12]. In warmer climates, complete ecdysis of unfed larvae to adult winter tick can occur within 20 days after infestation of a host [31,32]. The results of this study show the presence of both nymph and adult winter tick suggesting ticks with varying parasitic development rates. Disgorged winter tick nymph in diapause are found loosely in the hair of hosts. Programmed grooming by elk would remove these ticks and may account for our low number of nymph ticks found during collection [12,14,15,16]. It is possible that loose nymphs were lost during elk transport. However, we cannot speak to the probability this influenced our results as studies conducted similarly to ours found high numbers of nymph winter tick in hunter harvested deer [27]. Warmer temperatures may account for the presence of adult winter ticks in the fall months. Baer-Lehman noted the presence of adult winter ticks between November and December in Pennsylvania suggesting a shorter parasitic phase [27]. Studies of the winter tick life cycle in the northeast predate shifts in climate. To further understand winter tick parasitic phase in the northeast, more research is needed.

All 190 elk sampled in 2017 and 2018 showed no lesions related to winter tick infestations, potentially due to the low tick burdens that were identified. As described above, this may be a result of the life cycle of the winter tick and programmed grooming habits of elk [12]. Typically, winter tick related disease in moose, and sporadically other cervids, occur in the late winter through early spring [18,19,22]. Pregnancy in female elk coincide with winter tick activity. During this time, they may be more susceptible to the effects of weight loss that often accompany a severe winter tick infestation. Thorne found pregnant elk who lose at least 6.6% weight during pregnancy will bare smaller calves with poorer chances of survival [33]. To date, this has not been an observed problem on PA elk. Further monitoring is needed to better understand the affects winter ticks pose on pregnant elk.

Winter ticks can negatively impact the health of cervids indirectly through transmission of multiple pathogens. Tickborne diseases, such as *Babesia duncani*, *Francisella* like endosymbionts, *Borrelia burgdorferi*, *Anaplasma phagocytophilum*, and *Anaplasma marginale*, have been isolated from winter ticks [4,5,34,35]. Disease transmission between ticks is an additional risk of infestations and can occur in the absence of a systemic infection of the host via co-feeding transmission. Randolph described two types of co-feeding transmission between infected and uninfected ticks: (1) an uninfected tick feeds in close proximity to an infected tick simultaneously, and (2) by localized extended feeding where an uninfected tick feeds in the same location an infected tick has recently fed [36]. Preferential feeding of highly vascularized locations on a host increase the likelihood co-feeding transmission will occur. Although winter ticks are one host ticks, the risk for winter tick vertical transmission between hosts still exists due to programmed grooming habits of elk. Research suggests feeding ticks will find a new host when feeding is interrupted, creating the added risk for disease transmission between elk and other wildlife [4]. Moreover, *Dermacentor andersoni* was found to retain a high level of *A. marginale* infection after transmission to a host, suggesting the possibility for one tick to transmit disease to multiple hosts if removed during feeding [37]. To better understand the prevalence of disease in winter ticks of PA, more sampling and molecular analyses are needed.

## 5. Conclusions

The results of this study indicate the presence of two common tick species on wild elk during the fall months, namely blacklegged and winter tick. Winter tick infestations occur relatively frequently in elk; however, we found the burden in the fall is low potentially due to winter tick life cycle and elk grooming habits. While, winter ticks can have severe negative impacts on moose populations within the northeast, disease is rare in elk. However, with the ever-changing geographic expansion of ticks in the eastern US and the small localized population of wild elk in PA, continuous monitoring of winter ticks is warranted. A genetic analysis by Williams et al. characterized the Pennsylvania elk herd with high levels of inbreeding and low levels of unique and rare alleles [38]. This reduction in biological fitness makes the Pennsylvania elk population more susceptible to decline and possibly to pathogens. As described above, one limitation of this study is that it was restricted in the timing of sampling. Future studies should include tick collections during other seasons, especially spring and summer, to more completely characterize the tick species and abundance on Pennsylvania wild elk.

## Figures and Tables

**Table 1:** Total number of ticks collected from elk per county, Pennsylvania USA. Tick counts are based on the county in which elk were harvested. Data is organized by year, tick species, and county. A one-way ANOVA statistical analysis was performed to determine if there were differences between tick load per elk per county. A post hoc Tukey HSD analysis found differences represented by an asterisk in the table. An asterisk next to tick counts from 2017 and 2017/2018 indicate a difference to Elk county and an asterisk next to tick counts from 2018 indicate a difference to Cameron county. No location data was available for one elk. One deer tick collected from this elk was not included in the analysis.

Tick Counts per County									
County	2017			2018			Total (2017/2018)		
	Deer Tick	Winter Tick	Total Ticks	Deer Tick	Winter Tick	Total Ticks	Deer Tick	Winter Tick	Total Ticks
Elk	288	9	297	192 *	17	201 *	480	26	506
Clearfield	213	23	236 *	72 *	4	76 *	285	27	312
Cameron	218	15	223 *	125	1	126	343 *	16	359 *
Centre	62	11	73 *	60	4	64	122	15	137 *
Clinton	86	5	91 *	39	8	47	125	13	138

**Table 2:** Break-down of tick collection data separated by species and year. Average tick load, range per elk, total tick collections, tick life stage and sex are outlined for species and year. Overall, 1453 ticks were collected from a total of 190 elk during the 2017 (99 elk; 931 ticks) and 2018 (91 elk; 522 ticks) Pennsylvania elk hunt. The average tick load for both years combined was 7.7 with a range of 0–42 ticks per elk. The average winter tick load on all elk was 0.5 with a range of 0–9 winter ticks per elk.

	Deer Tick			Winter Tick			Total Ticks		
	2017	2018	2017–2018	2017	2018	Total	2017	2018	2017–2018
Tick Collections	868	488	1356	63	34	97	931	522	1453
Average Tick Load	8.8	5.4	7.1	0.6	0.4	0.5	9.4	5.7	7.7
Range of Ticks Per Elk	0–42	0–18	0–42	0–9	0–8	0–9	0–42	0–18	0–42
Total Adult Male	374	208	582	22	12	34	396	220	616
Total Adult Female	492	280	772	24	20	44	516	300	816
Total Nymphs	2	-	2	17	2	19	19	2	21
